# Clinical Challenge of Two Competing Targetable Mutations in Non-Small-Cell Lung Cancer: A Case Report

**DOI:** 10.3390/diagnostics13193112

**Published:** 2023-10-02

**Authors:** Sonya Youngju Park, Hyukjin Yoon, Eun Ji Han, Ie Ryung Yoo

**Affiliations:** Department of Radiology, College of Medicine, The Catholic University of Korea, Seoul 06591, Republic of Korea

**Keywords:** non-small-cell lung cancer, EGFR, ALK, FDG PET/CT

## Abstract

The development of therapeutic agents targeting products of epidermal growth factor receptor (EGFR) gene mutation and anaplastic lymphoma kinase (ALK) rearrangements has improved survival in patients with non-small-cell lung cancer. EGFR and ALK mutations are generally regarded as mutually exclusive, and the presence of one in lieu of another influences the response to targeted therapy. We herein present an interesting case following the course of progression of a patient with synchronous lung cancers with a discordant mutation profile. The importance of this modality in the follow-up of lung cancer patients is illustrated, and the therapeutic implications of coexisting oncogenic drivers are briefly discussed.

The recent update of the NCCN guidelines reflects the rise of precision medicine [1]. Molecular testing for specific mutations is recommended early on, and many targeted agents are now recommended as first-line therapy. Two of the key actionable oncogenic drivers in non-small-cell lung cancers are (1) EGFR mutations, which are the most frequently detected, being identified in 10% and 50% of cases in Western and Asian countries, respectively [2], and (2) ALK rearrangement, which occurs in approximately 5% of patients [3] (Supplementary Figure S1). EGFR mutations and ALK translocations are generally considered mutually exclusive, based on the heuristic model in which a single, early genomic driver event leads to a state of oncogene addiction, which drives tumorigenesis and tumor progression [4]. Therefore, the respective proteins are ideal targets for anticancer drugs, and treatment outcomes with these targeted therapies are superior to those achieved with conventional chemotherapy, in terms of improved progression-free survival and overall survival. However, a portion of NSCLCs, ranging from 1.3 to 1.6%, have been reported to have co-alterations [5,6], with increased rates when using more sensitive assays for EGFR, such as real-time PCR, targeted NGS or mutant-enriched NGS [7]. Two hypotheses have been proposed to explain the coexistence of multiple oncogenic drivers: (1) intratumoral heterogeneity with different genetic alterations occurring in different tumor cells and (2) multiple oncogenic pathways being altered in a single clone of tumor cells [8]. The limited reports on response to EGFR and/or ALK inhibitors in dual-positive patients have been variable [6,9,10,11,12,13], although it has been suggested that the relative activation status of EGFR and ALK, as determined by considering phosphorylation levels, could be predictive of inhibitor efficacy [6]. This is further complicated by the fact that EGFR mutations may contribute to resistance to ALK inhibitors [9], and vice versa [14], suggesting the need for combination therapy with EGFR –TKIs and ALK inhibitors.

Our case illustrates the variety of clinically important diagnostic and therapeutic problems posed by yet another challenge: synchronous lung cancers. Double primary tumors with discordant molecular biomarkers correspondingly result in a different profile of responses to target therapies, as further demonstrated by the serial F-18 FDG PET/CT images of our patient. In a small case series of non-small-cell lung cancer patients with monoclonal concomitant EGFR and ALK alterations, ALK inhibitors appeared to be effective for patients with co-alterations [7], but our patient eventually progressed while on lorlatinib. Given its rare occurrence, there is no consensus yet on the appropriate management of polyclonal (i.e., multiple) primary tumors with competing targetable mutations, and further studies are warranted to optimize the treatment strategies for synchronous lung cancers presenting with concomitant EGFR mutation and ALK translocation, reflecting the intricacies required for a more promising tailored approach for lung cancer patients.

**Figure 1:**
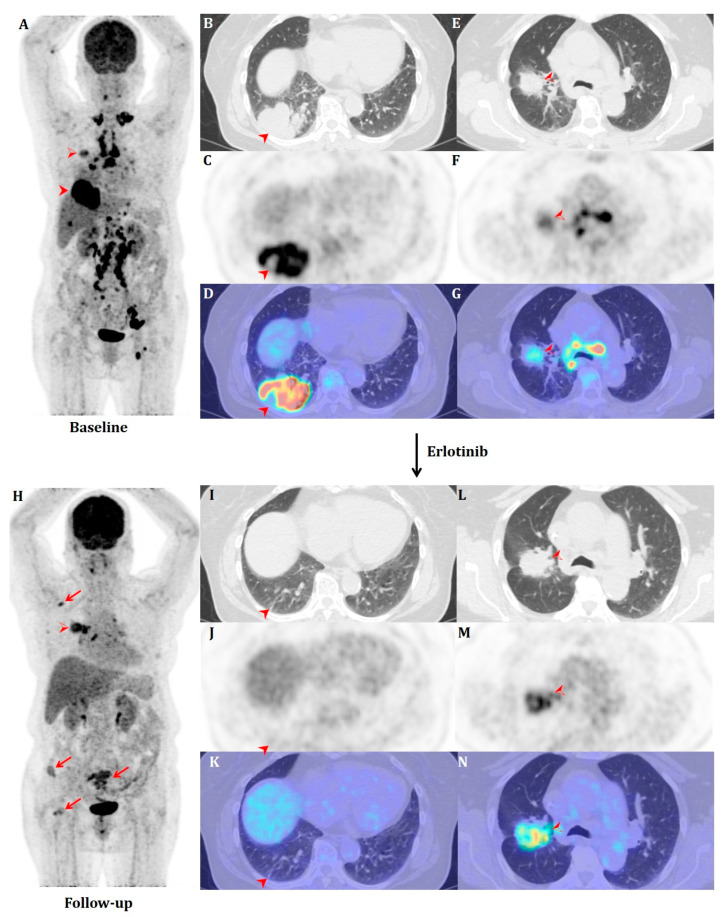
(**A**) 74-year-old nonsmoker female presenting with chronic cough was referred to our hospital for further evaluation and staging workup of two lung masses which were found on outside CT imaging: a 7.4 cm mass in the RLL abutting the pleura (indicated by arrowheads on (**B**), CT; (**C**), PET; and (**D**), fusion) and a 3.3 cm mass in the RUL with invasion of the right minor fissure (indicated by open arrowheads on (**E**), CT; (**F**), PET; and (**G**), fusion). Extensive metastatic lymphadenopathies were noted on both sides of the diaphragm, as well as liver and bone metastases (T4N3M1c) ((A), MIP). Cytologic diagnosis of bronchial washing of the LLL during bronchoscopy was non-small-cell lung cancer. PCNB of the larger mass proved to be a moderately differentiated adenocarcinoma, while mutation studies came back as EGFR(−)/ALK(+), and the patient was started on alectinib, the highly selective inhibitor of ALK. A subsequent response assessment showed marked regression, followed by stable disease on serial CT follow-up. However, restaging PET/CT a year later (**H**–**N**) showed that while the RLL mass was nearly resolved (**I**–**K**), the RUL mass (**L**–**N**) had in fact progressed, with several newly developed bone metastases in the right scapula, bilateral pelvic bones and right femur (indicated by red arrows on (**H**), MIP). An additional PCNB showed another moderately differentiated adenocarcinoma, but EGFR (+ for L858R missense mutation)/ALK(−), and the patient was switched to erlotinib, a classical EGFR tyrosine kinase inhibitor. Follow-up CT three months later showed marked regression of the RUL mass, but redevelopment of multiple abdominal nodes. The patient was then switched to lorlatinib to again cover the ALK rearrangement. Extrathoracic metastases showed complete response, but both primary tumors progressed two months later.

## Data Availability

The data presented in this study are available on request to the corresponding author. The data are not publicly available due to privacy/ethical reasons.

## Supplementary Materials

The following supporting information can be downloaded at: https://www.mdpi.com/article/10.3390/diagnostics13193112/s1, Figure S1: Molecular Mechanistm of EGFR mutations and ALK rearrangement.
