# Supplementary figures and images for: Clinical Challenge of Two Competing Targetable Mutations in Non-Small-Cell Lung Cancer: A Case Report
Source: Diagnostics (Basel). 2023 Oct 2;13(19):3112. doi: 10.3390/diagnostics13193112 (PMC10572277; doi:10.3390/diagnostics13193112)

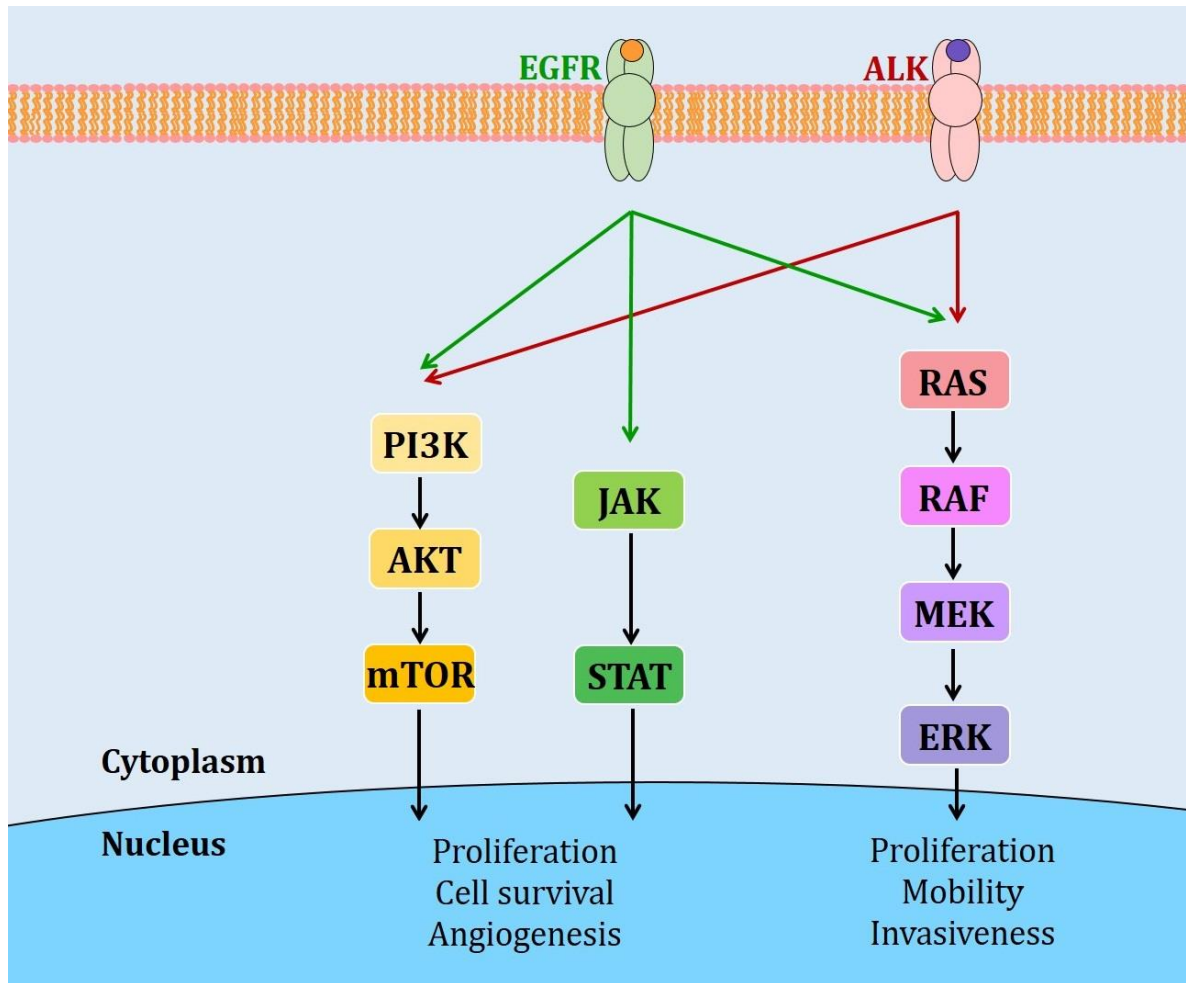

**Figure S1.** Molecular Mechanism of EGFR mutations and ALK rearrangement.

Supplement: Supplementary file 1 [file diagnostics-13-03112-s001.zip › diagnostics-2624994-supplementary.pdf]
